# 2-(2-Naphth­yl)-1,3-dioxane

**DOI:** 10.1107/S1600536810000644

**Published:** 2010-01-30

**Authors:** Damien Thevenet, Reinhard Neier, Helen Stoeckli-Evans

**Affiliations:** a Institute of Chemistry, University of Neuchâtel, rue Emile-Argand 11, 2009 Neuchâtel, Switzerland; b Institute of Physics, University of Neuchâtel, rue Emile-Argand 11, 2009 Neuchâtel, Switzerland

## Abstract

The title compound, C_14_H_14_O_2_, crystallizes in the chiral monoclinic space group *P*2_1_. This acetal is composed of a planar naphthalene ring with a 1,3-dioxane ring substituent, which has a chair conformation. In the crystal structure, symmetry-related mol­ecules are connected *via* a weak C—H⋯O inter­action to form a helical chain propagating in [010]. While there are no π–π stacking inter­actions present, there are weak C—H⋯π inter­actions involving the naphthalene aromatic rings, which link the helical chains to form a two-dimensional network in the (011) plane.

## Related literature

For information on commonly used protecting groups for carbonyl compounds, see: Kocienski (1994 ▶); Showler & Darley (1967 ▶). For methods for their deprotection, see: Cordes & Bull (1974 ▶); Fujioka *et al.* (2004 ▶); Ates *et al.* (2003 ▶). For kinetic and thermodynamic studies of acetals and ketals in the naphthalene series and other physical data, see: Newman & Dickson (1970 ▶); Carmichael & Hug (1986 ▶). For the synthesis of 2-naphthaldehyde acetal, see Gopinath *et al.* (2002 ▶). For details of the new photochemical reaction to hydrolyse the acetal into an aldehyde, see Thevenet & Neier (2010 ▶). For information on 1,3-dioxane ring related compounds, see: Buys & Eliel (1970 ▶). For the synthesis and crystal structure of a related compound, see: Borbas *et al.* (2002 ▶). For normal geometric parameters for mol­ecular compounds, see: Allen (2002 ▶).
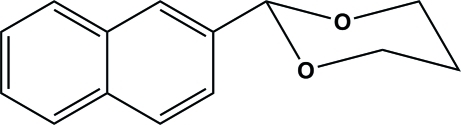


## Experimental

### 

#### Crystal data

C_14_H_14_O_2_
*M* *_r_* = 214.25
Monoclinic, 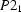

*a* = 7.5351 (6) Å
*b* = 7.8575 (8) Å
*c* = 9.4057 (9) Å
β = 92.839 (11)°
*V* = 556.20 (9) Å^3^
*Z* = 2
Mo *K*α radiation
μ = 0.08 mm^−1^
*T* = 173 K
0.38 × 0.30 × 0.08 mm

#### Data collection

Stoe IPDS diffractometer
4461 measured reflections
1098 independent reflections
951 reflections with *I* > 2σ(*I*)
*R*_int_ = 0.024

#### Refinement

*R*[*F* ^2^ > 2σ(*F* ^2^)] = 0.024
*wR*(*F* ^2^) = 0.061
*S* = 1.05
1098 reflections
145 parameters
1 restraint
H-atom parameters constrained
Δρ_max_ = 0.13 e Å^−3^
Δρ_min_ = −0.11 e Å^−3^

### 

Data collection: *EXPOSE* in *IPDS-I* (Stoe & Cie, 2000 ▶); cell refinement: *CELL* in *IPDS-I*; data reduction: *INTEGRATE* in *IPDS-I*; program(s) used to solve structure: *SHELXS97* (Sheldrick, 2008 ▶); program(s) used to refine structure: *SHELXL97* (Sheldrick, 2008 ▶); molecular graphics: *PLATON* (Spek, 2009 ▶) and *Mercury* (Macrae *et al.*, 2006 ▶); software used to prepare material for publication: *SHELXL97* and *PLATON*.

## Supplementary Material

Crystal structure: contains datablocks I, global. DOI: 10.1107/S1600536810000644/cv2685sup1.cif

Structure factors: contains datablocks I. DOI: 10.1107/S1600536810000644/cv2685Isup2.hkl

Additional supplementary materials:  crystallographic information; 3D view; checkCIF report

## Figures and Tables

**Table 1:** Hydrogen-bond geometry (Å, °) *Cg*1 and *Cg*2 are the centroids of the C1′–C4′/C9′/C10′ and C5′–C10′ rings, respectively.

*D*—H⋯*A*	*D*—H	H⋯*A*	*D*⋯*A*	*D*—H⋯*A*
C1′—H1′⋯O2^i^	0.95	2.60	3.349 (2)	136
C5′—H5′⋯*Cg*1^ii^	0.95	2.70	3.555 (2)	151
C4′—H4′⋯*Cg*2^ii^	0.95	2.92	3.776 (2)	150
C3—H3*A*⋯*Cg*1^i^	0.99	2.99	3.927 (2)	159

Symmetry codes: (i) 
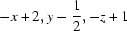
; (ii) 
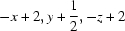
.

**Fig. 1.**
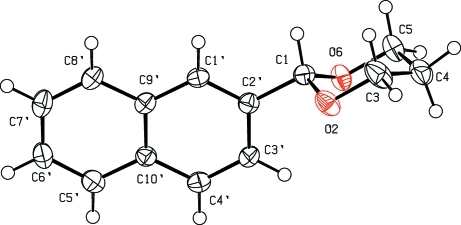
A view of the molecular structure of the title compound, with displacement ellipoids drawn at the 50% probabilty level.

**Fig. 2.**
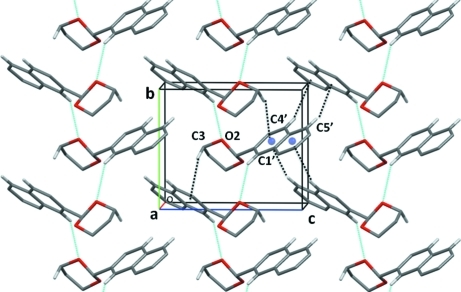
A view along the a axis of the crystal packing of the title compound. The C—H···O and C—H···π interactions are shown as dotted cyan and black lines, respectively. [The blue balls represent the centroids of the two aromatic rings; H-atoms not involved in the C—H···O and C—H···π interactions have been omitted for clarity; the C—H···π interactions are shown for one molecule only; see Table 1 for details].

## supplementary crystallographic information

### Comment

Acetals are the most commonly used protecting groups for carbonyl compounds in organic synthesis (Kocienski, 1994; Showler & Darley, 1967), and many methods have been developed for their deprotection (Cordes & Bull, 1974; Fujioka *et al.*, 2004; Ates *et al.*, 2003). The title 2-naphthaldehyde acetal (Newman & Dickson, 1970; Carmichael & Hug, 1986) was synthesized to investigate the scope of a new photochemical reaction capable of hydrolysing the acetal into an aldehyde (Thevenet & Neier, 2010). The NMR spectra of the unsubstituted 1,3-dioxane ring displays a complicated AA'BB'MN system (Buys & Eliel, 1970), and the X-ray crystal structure was helpful for the interpretation of the NMR spectra (Thevenet & Neier, 2010).

The structure of the title compound is illustrated in Fig. 1, and the geometrical parameters are given in the Supplementary information and the archived CIF. The bond lengths and angles are close to those in three similar compounds located in the Cambridge Crystal Structure Database (CSD, V 5.30, last update Sept. 2009; Allen, 2002). For example, methyl 2,3-di-*O*-acteyl-4,6-*O*-(2-naphthyl)methylene-α-*D*-galactopyranoside (Borbas *et al.*, 2002), which also crystallized in the monoclinic space group *P*2_1_, and where the naphthalene ring is planar and the two six-membered rings in the galactopyranoside unit have chair conformations.

In the crystal of the title compound symmetry related molecules are connected *via* a C—H···O interaction (Table 1) giving rise to the formation of helical chains propagating in [010]. These chains are further linked *via* weak C—H···π interactions to form a two-dimensional network in (011) - see Fig. 2 and Table 1 for details.

### Experimental

The title compound was synthesized using a modified strategy described by (Gopinath *et al.*, 2002). To a solution of 2-naphthaldehyde (0.64 mmol), trimethylorthoformate (1.41 mmol) and 1,3-propanediol (5.12 mmol) in dry nitromethane (2 ml) was added tetrabutylammonium tribromide (0.025 mmol). The homogeneous reaction mixture was stirred at r.t. and the progress of the reaction monitored by TLC and GC. After completion of the reaction the mixture was poured into a solution of NaHCO_3_ (10 ml) and the products were extracted with diethyl ether (3 × 10 ml). The organic layer was separated, dried over anhydrous Na_2_SO_4_ and concentrated. The white solid obtained was purified by recrystallization in MeOH, giving colourless thin plate-like crystals of the title compound.

^1^H NMR 400 MHz (CDCl_3_) δ 7.97 (br s, 1H, H_1'_), 7.85 (m, 3H, H_4',5',8'_), 7.60 (dd, 1H, ^3^J_3'-4'_ = 8.5 Hz, ^3^J_3'-1'_ = 1.7 Hz, H_3'_), 7.48 (m, 2H, H_6',7'_), 5.68 (s, 1H, H_1_), 4.33 (dddd, 2H, ^2^J_3e-3a;5e-5a_ = -11.7 Hz, ^3^J_3e-4a;5e-4a_ = 5.0 Hz, ^3^J_3e-4e;5e-4e_ = 1.5 Hz, ^4^J_3e-5e_ = 3.0 Hz, H_3e,5e_), 4.06 (ddd, 2H, ^2^J_3a-3e;5a-5e_ = -11.7 Hz, ^3^J_3a-4a;5a-4a_ = 12.4 Hz, ^3^J_3a-4e;5a-4e_ = 2.7 Hz, H_3a,5a_), 2.29( dtt, 1H, ^2^J_4a-4e_ = -13.5 Hz, ^3^J_4a-3a;4a-5a_ = 12.4 Hz, ^3^J_4a-3e;4a-5e_ = 5.0 Hz, H_4a_), 1.50 (dtt, 1H, ^2^J_4e-4a_ = -13.5 Hz, ^3^J_4e-3a;4e-5a_ = 2.7 Hz, ^3^J_4e-3e;4e-5e_ = 1.5 Hz, H_4 e_); ^13^C NMR 100 MHz (CDCl_3_) δ 136.1 (C_2'_), (133.6, 133.1) (C_9',10'_), (128.4, 128.1, 127.7) (C_4',5',8'_), (126.2, 126.0) (C_6',7'_), 125.3 (C_1'_), 123.8 (C_3'_), 101.8 (C_1_), 67.5 (C_3,5_), 25.9 (C_4_); HRMS (ESI, +): [*M* + Na]^+^ = 237.09. Note: The same numbering scheme has been used for the crystal structure (Fig. 1). The torsional angles of the 1,3-dioxane ring were measured to estimate the coupling constants according to the Karplus equation.

### Refinement

In the final cycles of refinement, in the absence of significant anomalous scattering effects, 944 (93%) Friedel pairs were merged and Δf" set to zero. The H-atoms could all be located in difference electron-density maps. In the final cycles of refinement they were included in calculated positions and treated as riding atoms: C—H = 0.95–1.0 Å, with *U*_iso_(H) = 1.2*U*_eq_(parent C-atom). Using the one-circle Stoe Image Plate Diffraction System it is not always possible to measure 100% of the Ewald sphere, and here only 93.7% of the data were accessible out to 50° in 2θ. This has little effect on the bond distances and angles when comparing their values with those of the related structure mentioned above (Borbas *et al.*, 2002).

### Crystal data

**Table:**

C_14_H_14_O_2_	*F*(000) = 228
*M**_r_* = 214.25	*D*_x_ = 1.279 Mg m^−^^3^
Monoclinic, *P*2_1_	Mo *K*α radiation, λ = 0.71073 Å
Hall symbol: P 2yb	Cell parameters from 4553 reflections
*a* = 7.5351 (6) Å	θ = 2.1–26.0°
*b* = 7.8575 (8) Å	µ = 0.08 mm^−^^1^
*c* = 9.4057 (9) Å	*T* = 173 K
β = 92.839 (11)°	Plate, colourless
*V* = 556.20 (9) Å^3^	0.38 × 0.30 × 0.08 mm
*Z* = 2	

### Data collection

**Table:**

Stoe IPDS diffractometer	951 reflections with *I* > 2σ(*I*)
Radiation source: fine-focus sealed tube	*R*_int_ = 0.024
graphite	θ_max_ = 26.0°, θ_min_ = 2.2°
φ rotation scans	*h* = −8→8
4461 measured reflections	*k* = −9→9
1098 independent reflections	*l* = −11→11

### Refinement

**Table:**

Refinement on *F*^2^	Primary atom site location: structure-invariant direct methods
Least-squares matrix: full	Secondary atom site location: difference Fourier map
*R*[*F*^2^ > 2σ(*F*^2^)] = 0.024	Hydrogen site location: inferred from neighbouring sites
*wR*(*F*^2^) = 0.061	H-atom parameters constrained
*S* = 1.05	*w* = 1/[σ^2^(*F*_o_^2^) + (0.0412*P*)^2^] where *P* = (*F*_o_^2^ + 2*F*_c_^2^)/3
1098 reflections	(Δ/σ)_max_ < 0.001
145 parameters	Δρ_max_ = 0.13 e Å^−^^3^
1 restraint	Δρ_min_ = −0.11 e Å^−^^3^

### Special details

**Table:**

Geometry. Bond distances, angles *etc*. have been calculated using the rounded fractional coordinates. All su's are estimated from the variances of the (full) variance-covariance matrix. The cell e.s.d.'s are taken into account in the estimation of distances, angles and torsion angles
Refinement. In the final cycles of refinement, in the absence of significant anomalous scattering effects, 944 (93%) Friedel pairs were merged and Δf " set to zero. The H-atoms could all be located in difference electron-density maps. In the final cycles of refinement they were included in calculated positions and treated as riding atoms: C—H = 0.95 - 1.0 Å, with *U*_iso_(H) = 1.2*U*_eq_(parent C-atoms). Using the one-circle Stoe Image Plate Diffraction System it is not always possible to measure 100% of the Ewald sphere, and here only 93.7% of the data were accessible out to 50° in 2θ.

### Fractional atomic coordinates and isotropic or equivalent isotropic displacement parameters (Å^2^)

**Table:**

	*x*	*y*	*z*	*U*_iso_*/*U*_eq_	
O2	1.16506 (19)	0.57058 (16)	0.43163 (12)	0.0337 (4)	
O6	1.33418 (18)	0.38156 (15)	0.57014 (12)	0.0295 (4)	
C1	1.1616 (2)	0.4392 (2)	0.53276 (17)	0.0251 (5)	
C1'	0.9100 (2)	0.46504 (19)	0.69218 (16)	0.0239 (5)	
C2'	1.0796 (2)	0.5075 (2)	0.66364 (16)	0.0240 (5)	
C3	1.2305 (3)	0.5057 (3)	0.30168 (18)	0.0417 (7)	
C3'	1.1796 (3)	0.6163 (2)	0.75664 (17)	0.0277 (6)	
C4	1.4131 (3)	0.4321 (3)	0.32815 (19)	0.0400 (7)	
C4'	1.1073 (3)	0.6764 (2)	0.87667 (18)	0.0297 (6)	
C5	1.4128 (3)	0.3067 (3)	0.44944 (18)	0.0348 (6)	
C5'	0.8555 (3)	0.6908 (2)	1.03543 (18)	0.0309 (6)	
C6'	0.6865 (3)	0.6467 (2)	1.06380 (18)	0.0316 (6)	
C7'	0.5852 (3)	0.5438 (2)	0.96939 (18)	0.0328 (6)	
C8'	0.6551 (2)	0.4853 (2)	0.84779 (18)	0.0284 (5)	
C9'	0.8310 (2)	0.52609 (19)	0.81562 (16)	0.0236 (5)	
C10'	0.9334 (2)	0.63231 (19)	0.91039 (17)	0.0239 (5)	
H1	1.08840	0.34250	0.49320	0.0300*	
H1'	0.84350	0.39320	0.62810	0.0290*	
H3'	1.29720	0.64750	0.73540	0.0330*	
H3A	1.14900	0.41670	0.26250	0.0500*	
H3E	1.23480	0.59870	0.23090	0.0500*	
H4'	1.17550	0.74940	0.93860	0.0360*	
H4A	1.45060	0.37410	0.24110	0.0480*	
H4E	1.49890	0.52450	0.35160	0.0480*	
H5'	0.92210	0.76160	1.10020	0.0370*	
H5A	1.53630	0.27170	0.47590	0.0420*	
H5E	1.34500	0.20400	0.41910	0.0420*	
H6'	0.63660	0.68620	1.14860	0.0380*	
H7'	0.46700	0.51460	0.99020	0.0390*	
H8'	0.58470	0.41640	0.78400	0.0340*	

### Atomic displacement parameters (Å^2^)

**Table:**

	*U*^11^	*U*^22^	*U*^33^	*U*^12^	*U*^13^	*U*^23^
O2	0.0500 (9)	0.0289 (6)	0.0225 (6)	0.0088 (6)	0.0049 (5)	0.0012 (5)
O6	0.0283 (8)	0.0376 (7)	0.0228 (5)	0.0058 (6)	0.0027 (5)	−0.0007 (5)
C1	0.0269 (11)	0.0239 (8)	0.0244 (8)	−0.0013 (6)	0.0008 (7)	0.0000 (6)
C1'	0.0245 (11)	0.0228 (8)	0.0240 (8)	−0.0016 (6)	−0.0023 (7)	0.0007 (6)
C2'	0.0265 (11)	0.0229 (8)	0.0225 (8)	−0.0003 (7)	0.0012 (7)	0.0021 (7)
C3	0.0679 (17)	0.0358 (9)	0.0220 (8)	0.0095 (10)	0.0084 (9)	0.0013 (8)
C3'	0.0224 (11)	0.0303 (9)	0.0307 (9)	−0.0050 (7)	0.0031 (7)	−0.0032 (7)
C4	0.0533 (16)	0.0374 (10)	0.0306 (9)	−0.0015 (9)	0.0158 (9)	−0.0061 (8)
C4'	0.0273 (12)	0.0299 (9)	0.0317 (9)	−0.0050 (7)	−0.0007 (7)	−0.0058 (7)
C5	0.0371 (13)	0.0395 (10)	0.0283 (9)	0.0069 (8)	0.0077 (8)	−0.0053 (8)
C5'	0.0345 (14)	0.0286 (9)	0.0298 (9)	0.0016 (7)	0.0026 (8)	−0.0023 (7)
C6'	0.0322 (12)	0.0327 (9)	0.0309 (8)	0.0066 (8)	0.0105 (7)	0.0024 (7)
C7'	0.0230 (12)	0.0382 (11)	0.0377 (9)	0.0031 (7)	0.0063 (8)	0.0075 (8)
C8'	0.0222 (11)	0.0316 (9)	0.0313 (8)	−0.0035 (8)	0.0004 (7)	0.0029 (7)
C9'	0.0222 (11)	0.0226 (8)	0.0257 (8)	0.0007 (6)	−0.0005 (7)	0.0051 (6)
C10'	0.0242 (11)	0.0211 (7)	0.0264 (8)	0.0010 (7)	0.0013 (7)	0.0007 (6)

### Geometric parameters (Å, °)

**Table:**

O2—C1	1.405 (2)	C8'—C9'	1.411 (2)
O2—C3	1.434 (2)	C9'—C10'	1.421 (2)
O6—C1	1.405 (2)	C1—H1	1.0000
O6—C5	1.433 (2)	C1'—H1'	0.9500
C1—C2'	1.504 (2)	C3—H3A	0.9900
C1'—C2'	1.360 (2)	C3—H3E	0.9900
C1'—C9'	1.415 (2)	C3'—H3'	0.9500
C2'—C3'	1.414 (2)	C4—H4A	0.9900
C3—C4	1.502 (3)	C4—H4E	0.9900
C3'—C4'	1.362 (3)	C4'—H4'	0.9500
C4—C5	1.508 (3)	C5—H5A	0.9900
C4'—C10'	1.407 (3)	C5—H5E	0.9900
C5'—C6'	1.359 (3)	C5'—H5'	0.9500
C5'—C10'	1.417 (2)	C6'—H6'	0.9500
C6'—C7'	1.399 (3)	C7'—H7'	0.9500
C7'—C8'	1.363 (2)	C8'—H8'	0.9500
			
C1—O2—C3	109.55 (14)	C9'—C1'—H1'	119.00
C1—O6—C5	110.36 (13)	O2—C3—H3A	110.00
O2—C1—O6	110.99 (13)	O2—C3—H3E	110.00
O2—C1—C2'	108.32 (13)	C4—C3—H3A	110.00
O6—C1—C2'	108.84 (13)	C4—C3—H3E	110.00
C2'—C1'—C9'	121.09 (14)	H3A—C3—H3E	108.00
C1—C2'—C1'	120.15 (14)	C2'—C3'—H3'	120.00
C1—C2'—C3'	119.63 (14)	C4'—C3'—H3'	120.00
C1'—C2'—C3'	120.22 (15)	C3—C4—H4A	110.00
O2—C3—C4	110.27 (15)	C3—C4—H4E	110.00
C2'—C3'—C4'	119.98 (19)	C5—C4—H4A	110.00
C3—C4—C5	109.96 (18)	C5—C4—H4E	110.00
C3'—C4'—C10'	121.12 (17)	H4A—C4—H4E	108.00
O6—C5—C4	110.33 (18)	C3'—C4'—H4'	119.00
C6'—C5'—C10'	120.74 (16)	C10'—C4'—H4'	119.00
C5'—C6'—C7'	120.65 (17)	O6—C5—H5A	110.00
C6'—C7'—C8'	120.42 (19)	O6—C5—H5E	110.00
C7'—C8'—C9'	120.64 (16)	C4—C5—H5A	110.00
C1'—C9'—C8'	122.48 (14)	C4—C5—H5E	110.00
C1'—C9'—C10'	118.47 (14)	H5A—C5—H5E	108.00
C8'—C9'—C10'	119.06 (14)	C6'—C5'—H5'	120.00
C4'—C10'—C5'	122.42 (15)	C10'—C5'—H5'	120.00
C4'—C10'—C9'	119.09 (14)	C5'—C6'—H6'	120.00
C5'—C10'—C9'	118.49 (15)	C7'—C6'—H6'	120.00
O2—C1—H1	110.00	C6'—C7'—H7'	120.00
O6—C1—H1	110.00	C8'—C7'—H7'	120.00
C2'—C1—H1	110.00	C7'—C8'—H8'	120.00
C2'—C1'—H1'	119.00	C9'—C8'—H8'	120.00
			
C3—O2—C1—O6	64.83 (17)	O2—C3—C4—C5	51.9 (2)
C3—O2—C1—C2'	−175.74 (14)	C2'—C3'—C4'—C10'	−0.1 (3)
C1—O2—C3—C4	−58.4 (2)	C3—C4—C5—O6	−50.8 (2)
C5—O6—C1—O2	−64.14 (17)	C3'—C4'—C10'—C5'	179.07 (16)
C5—O6—C1—C2'	176.74 (14)	C3'—C4'—C10'—C9'	−1.3 (2)
C1—O6—C5—C4	56.5 (2)	C10'—C5'—C6'—C7'	−0.6 (3)
O2—C1—C2'—C1'	104.47 (17)	C6'—C5'—C10'—C4'	179.42 (16)
O2—C1—C2'—C3'	−75.40 (18)	C6'—C5'—C10'—C9'	−0.2 (2)
O6—C1—C2'—C1'	−134.75 (15)	C5'—C6'—C7'—C8'	0.4 (3)
O6—C1—C2'—C3'	45.38 (19)	C6'—C7'—C8'—C9'	0.6 (2)
C9'—C1'—C2'—C1	179.19 (14)	C7'—C8'—C9'—C1'	178.60 (15)
C9'—C1'—C2'—C3'	−0.9 (2)	C7'—C8'—C9'—C10'	−1.4 (2)
C2'—C1'—C9'—C8'	179.59 (15)	C1'—C9'—C10'—C4'	1.6 (2)
C2'—C1'—C9'—C10'	−0.5 (2)	C1'—C9'—C10'—C5'	−178.80 (14)
C1—C2'—C3'—C4'	−178.90 (15)	C8'—C9'—C10'—C4'	−178.48 (15)
C1'—C2'—C3'—C4'	1.2 (2)	C8'—C9'—C10'—C5'	1.2 (2)

### Hydrogen-bond geometry (Å, °)

**Table:**

*D*—H···*A*	*D*—H	H···*A*	*D*···*A*	*D*—H···*A*
C1'—H1'···O2^i^	0.95	2.60	3.349 (2)	136
C5'—H5'···Cg1^ii^	0.95	2.70	3.555 (2)	151
C4'—H4'···Cg2^ii^	0.95	2.92	3.776 (2)	150
C3—H3A···Cg1^i^	0.99	2.99	3.927 (2)	159

Symmetry codes: (i) −*x*+2, *y*−1/2, −*z*+1; (ii) −*x*+2, *y*+1/2, −*z*+2.
